# A hybrid breast cancer classification algorithm based on meta-learning and artificial neural networks

**DOI:** 10.3389/fonc.2022.1042964

**Published:** 2022-11-11

**Authors:** Luyao Han, Zhixiang Yin

**Affiliations:** Center of Intelligent Computing and Applied Statistics, School of Mathematics, Physics and Statistics, Shanghai University of Engineering Science, Shanghai, China

**Keywords:** breast cancer, feature selection, machine learning algorithm, meta-learning, ANN

## Abstract

The incidence of breast cancer in women has surpassed that of lung cancer as the world’s leading new cancer case. Regular screening and measures become an effective way to prevent breast cancer and also provide a good foundation for later treatment. Women should receive regular checkups in the hospital after reaching a certain age. The use of computer-aided technology can improve the accuracy and efficiency of physicians’ decision-making. Data pre-processing is required before data analysis, and 16 features are selected using a correlation-based feature selection method. In this paper, meta-learning and Artificial Neural Networks (ANN) are combined to create a hybrid algorithm. The proposed hybrid algorithm for predicting breast cancer was attempted to achieve 98.74% accuracy and 98.02% F1-score by creating a combination of various meta-learning models whose output was used as input features for creating ANN models. Therefore, the hybrid algorithm proposed in this paper can obtain better prediction results than a single model.

## Introduction

Cancer is one of the diseases that can pose a serious threat to human life and social development. As a whole, cancer is the second leading cause of death worldwide. In the past few decades, good progress has been made in basic research and clinical treatment of cancer, but it also leaves doctors at their wits’ end to cure advanced cancer. The prevention and early treatment of cancer is a great challenge to health problems. The top cancers in terms of global incidence are breast cancer, lung cancer, colorectal cancer, prostate cancer, and stomach cancer. The most common cancer in women worldwide is breast cancer, which is also the leading cause of death in women (1, 2). Early screening, early prevention, and early diagnosis can increase the likelihood of an effective cure for breast cancer. Women over the age of 40 should visit the hospital regularly for breast screening, which is still diagnosed at a late stage due to women’s negligence in breast self-examination and clinical examination. The main common screening modalities for breast cancer are mammography, radiography, ultrasound, and magnetic resonance imaging (MRI) (3, 4). Radiography is widely used in the diagnosis of breast cancer because it is less invasive and has fewer complications in clinical use. Radiography is prone to false-positive and false-negative results in detecting breast cancer (5), so in this study, we used a breast cancer diagnosis from the Wisconsin Diagnostic Breast Cancer (WDBC), where medical personnel acquired digital images of a patient’s breast mass after fine needle aspiration (FNA) and extracted features from these digital images that describe the presentation of cell nuclei in the images.

Various deep learning studies and machine learning techniques are used for the classification and identification of breast cancer types. The development of deep learning techniques has an important role in improving the diagnostic performance of breast cancer and expanding its clinical applications. Soham Chattopadhyay et al. (6) proposed a deep learning model dense residual dual-shuffle attention network (DRDA-Net) was added with a channel attention mechanism to the deep learning model DRDA-Net, which can greatly improve the model’s ability to learn complex patterns of images. Although the model was implemented with a small BreakHis dataset, the densely connected blocks of the model solved the problems of overfitting and hourly gradients well and finally achieved a classification accuracy of up to 98.1%. Chatterjee S et al. (7) proposed a two-stage deep learning model for breast cancer detection in thermal imaging images. Firstly, features were extracted from the images using VGG16, and secondly, a modified Dragonfly Algorithm (DA) meta-heuristic was used and the proposed two-stage framework achieved 100% diagnostic accuracy on a small dataset DMR-IR. Recent studies have shown that deep learning is widely used in breast tissue identification. Sneider A et al. (8) used a deep learning channel based on a convolutional neural network (CNN) trained on H&E stained slides near breast tumors and tissues to successfully identify and classify seven cell and tissue classes in 32 patient tissue samples. The overall test accuracy was 93.0%. Khairnar S et al. (9) used mammogram images from the MIAS database and applied various image binarization methods to extract image features such as OTSU, Niblack, Bernsen, Thepade’s Sorted Block Truncation (TSBTC), where TSBTC is applied for the first time in breast cancer identification. The features are input to machine learning algorithms for classifying tumor types as benign, malignant, and normal tumors, and finally for breast cancer identification.

Machine learning can be applied in the pharmaceutical industry to diagnose cancer (10, 11). As artificial intelligence continues to be applied to the medical field, the accuracy and speed of physicians’ decisions have improved. The application of ML models can improve the quality of medical data, save medical costs and help doctors to improve decision-making (12). ML is divided into supervised and unsupervised, ML can be used to diagnose whether a mass is benign or malignant, and ML can be evaluated by accuracy, precision, recall, and F1-score (12, 13). Different machine learning algorithms have different prediction accuracy, and ensemble techniques can solve this problem. Integration methods can improve the prediction ability of weak learners by combining several weak learners into one strong learner. Integration techniques can achieve the following effects: Bagging, Boosting, or Stacking (10). This study focuses on the stacking approach, which combines multiple machine learning models into one strong classification model. It combines both Bagging and Boosting integration methods to largely improve the machine learning prediction effect. It uses meta-learning algorithms to learn how best to combine predictions from two or more basic machine learning algorithms such as KNN, SVM, and DT. Meta-learning is the ability to “learn to learn” like humans (14). In meta-learning, each model is trained with a different set of training tasks and such models are combined to form a body of knowledge that is applied to a new unknown task and the results are analyzed (15). Ghiasi Mohammad M et al. (16) used ensemble learning based on decision trees to classify breast cancer and obtained 100% accuracy for breast cancer type classification using RF and ET based on the WBCD dataset. M. S. K. Inan (17) integrated three machine learning algorithms of logistic regression, support vector machine and K-nearest neighbor used to predict breast cancer classification and obtained an accuracy of 98.25%. A. Bharat et al. (1) compared four machine learning algorithms, Support Vector Machine (SVM), Decision Tree (C4.5), Naive Bayes (NB), and K-nearest neighbors (K-nn) on Wisconsin Diagnostic Breast Cancer, SVM gave the highest accuracy of 97.13%. Karthik et al. (18) used Recursive Feature Elimination (RFE) followed by Deep Neural Network (DNN) as the classifier model with 98.62% accuracy. Kourou K (19) used different ML methods to complete the classification task on breast cancer data by feature selection to extract important information from the dataset. S. M. S et al. (20) used the logistic regression technique to classify predicted breast cancer obtaining 96.5%.

In this paper, two main parts are included: feature engineering and classification. The feature engineering part first removes the features with high relevance in the form of a heat map, and the remaining features are selected using relevance-based feature selection, recursive feature elimination with cross-validation (REFCV), and tree-based feature selection. The accuracy is verified in the form of random forest classification and 16 features are obtained by relevance-based feature selection. The classification algorithm requires nine machine learning algorithms to construct a meta-learning framework, and the above features are input to the meta-learning framework, and their output is input to the ANN model to obtain a good classification of benign and malignant cases.

The second part of this paper introduces the relevant theoretical knowledge and operation steps, the third part introduces the obtained results, and the fourth part summarizes the paper and puts forward the prospect for the future development.

## Materials and methods

In this paper, we use a hybrid approach of meta-learning and ANN to diagnose breast cancer. The paper mainly includes feature engineering and prediction models. First, the data is preprocessed and a relevance-based feature selection method is selected to extract the most important features (19). Secondly, the meta-learning method learns the excellent performance of various machine learning algorithms, such as SVM, KNN, LR, etc. The learning results are input to the ANN model and 98.74% accuracy is obtained.

### Data description

**Figure 1:**
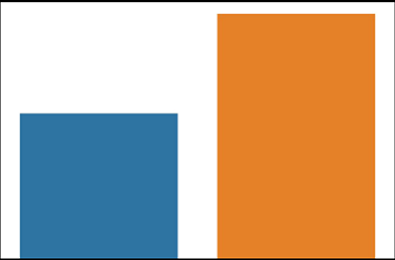
Distribution of benign and malignant cases in the dataset (M=malignant, B=benign).

As shown in **Table 1**, we use the WDBC, which has 569 samples and 30 features extracted from cell nuclear biopsy images, which are three dimensions of ten features: mean, standard deviation, and maximum. The ratio of benign to malignant in the dataset shown in **Figure 1** is B: M=357: 212. This dataset is commonly used in breast cancer prediction to determine whether a tumor is malignant or benign. The split ratio of the training set and testing set in the dataset is 70:30.

**Table 1:** Data description.

SN	Attributes	Description
1	Radius	Mean of distance from the center to points on the perimeter
2	Texture	The standard deviation of gray-scale values
3	Perimeter	The outer perimeter of the tumor in the image
4	Area	Tumor image covered with content
5	Smoothness	Local variation in radius lengths
6	Compactness	Perimeter^2/area-1.0
7	Concavity	The severity of concave portions of the contour
8	Concave points	Number of concave portions of the contour
9	Symmetry	Image two sides symmetry
10	Fractal dimension	Coastline approximation-1

### Feature engineering

#### Pre-processing data

Data pre-processing is an important stage in machine learning algorithms (21). First, the data are normalized and the processed data are subjected to feature selection and classification prediction. Secondly, to solve the unbalanced problem of data, the Synthetic Minority Over-Sampling Technique (SMOTE) was adopted.

A. Data normalization is the processing of data so that the mean of the values in each feature becomes 0 and the standard deviation becomes 1 (22).


$$\begin{array}{c} x^{*}=\frac{x_{i}-mean\left(x\right)}{st\left(x\right)}(1) \end{array}$$

B. SMOTE is commonly used to address data category imbalance, and we used the SMOTE technique to address the risk of a few malignant cases in our sample (17).

#### Feature selection

In this study, we will select features with different methods that are feature selection with correlation, RFECV, and tree-based feature selection. We will use random forest classification to train our model and predict.

**Figure 2:**
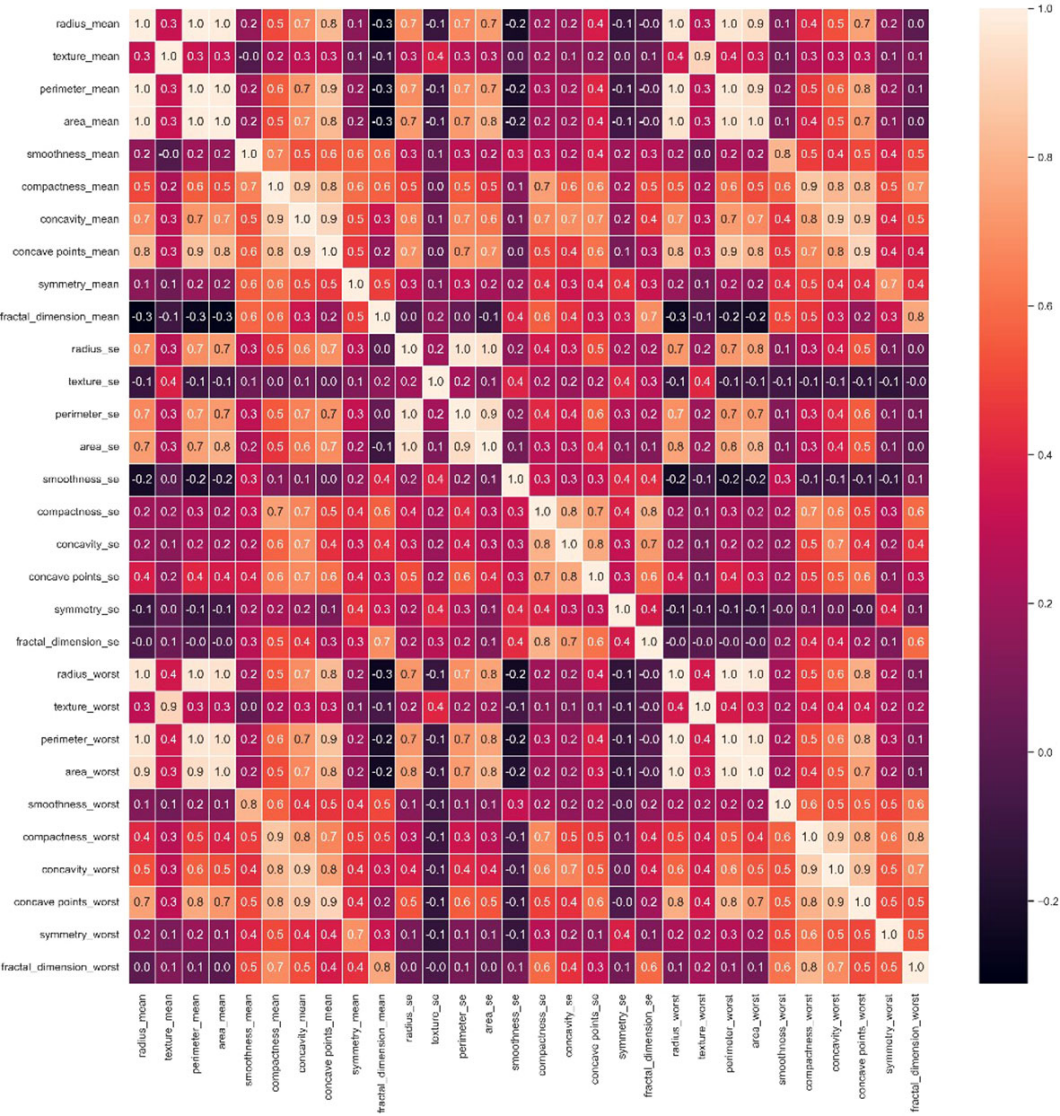
Breast cancer correlation heatmap.

The diagonal in the heat map **Figure 2** is the correlation coefficient of the univariate itself is 1. The lighter color represents a higher correlation (23, 24). From here we have to reduce the interlinked feature. If we don’t drop those interlinked columns and just put one column from those columns into our final data frame, then it will cause multi-correlation, which will reduce the accuracy of the model. For example, radius_mean is linearly related to perimeter_mean, perimeter_mean is linearly related to area_mean, and area_mean is also linearly dependent on radius_mean. So radius_mean, perimeter_mean and area_mean are interlinked. And we will drop perimeter_mean and radius_mean. In this study, we have selected 16 attribute variables as representatives.

### Machine learning algorithm

In this study, multiple types of machine learning techniques were used for the classification prediction of breast cancer, including strong classifiers and weak classifiers.

#### SVM

SVM is a classification model that belongs to the supervised algorithm (17). The main idea is to be able to separate hyperplanes at intervals, maximizing while correctly dividing the training set (19, 25). SVM can effectively solve high-dimensional feature classification regression problems and is widely used in classification problems (26).

#### KNN

KNN is one of the simplest non-parametric classification methods (17, 21). The principle of KNN is that when a new value x is predicted, the class of x is determined based on what class its nearest K points belong to (27, 28). The value of K is chosen as appropriate based on cross-validation. The common way of measuring distance in the algorithm is the Euclidean distance, which is calculated using.


$$\begin{array}{c} d(x,y)=\sqrt{\sum_{i=1}^{n}{\left(x_{i}-y_{i}\right)}^{2}}\left(2\right) \end{array}$$

where x and y are the features for which distances need to be calculated.

#### DT

The decision tree is a supervised machine learning algorithm (27). A decision tree usually consists of a root node, multiple internal nodes, and leaf nodes, where the root node contains all samples, the leaf nodes correspond to decision outcomes, and the other nodes correspond to an attribute test (23). The decision tree is a process of dividing the full set of samples from the root node to the child nodes based on the attribute tests. The simple structure and ease of representation are the main advantages of decision trees.

#### RF

Random forest is an integrated algorithm consisting of decision trees (26, 28). When performing a classification task, each decision tree is allowed to judge and classify individually, and the result that gets the most out of all decision trees is taken as the final result. The advantages of random forests include less susceptibility to overfitting and faster training, which makes them widely used in classification tasks.

#### LR

LR is a classification model commonly used for binary classification. The term “regression” in logistic regression refers to the return of a value to a value between 0 and 1 (23). Logistic regression is a simple and easy-to-interpret model that is commonly used to classify data.

#### GB

GB is an integrated model based on decision trees (26). The GB algorithm improves the performance of the algorithm by fitting negative gradients.

#### XGB

XGBoost is an ensemble algorithm based on DT (17). XGBoost continuously learns the DT, adding regularization terms and minimizing the loss function.

#### Adaboost

Adaboost is one of the boosting algorithms. Adaboost takes the weighted set of weak classifiers and makes it a strong classifier (26). During the training process, Adaboost increases the weights of samples that were classified incorrectly by the previous round of classifiers and decreases the weights of samples that were classified correctly. By increasing the weights of the weak classifiers with small classification error rates and continuously iterating, the overall classification error rate is improved.

#### MLP

Multilayer perceptron (MLP) is a neural network-based classification algorithm. The bottom layer of MLP is the input layer, the middle layer is the hidden layer, and finally the output layer. The different layers of the MLP neural network are fully connected (any neuron of the upper layer is connected to all neurons of the lower layer) (23, 26, 27).

### Proposed method

**Figure 3:**
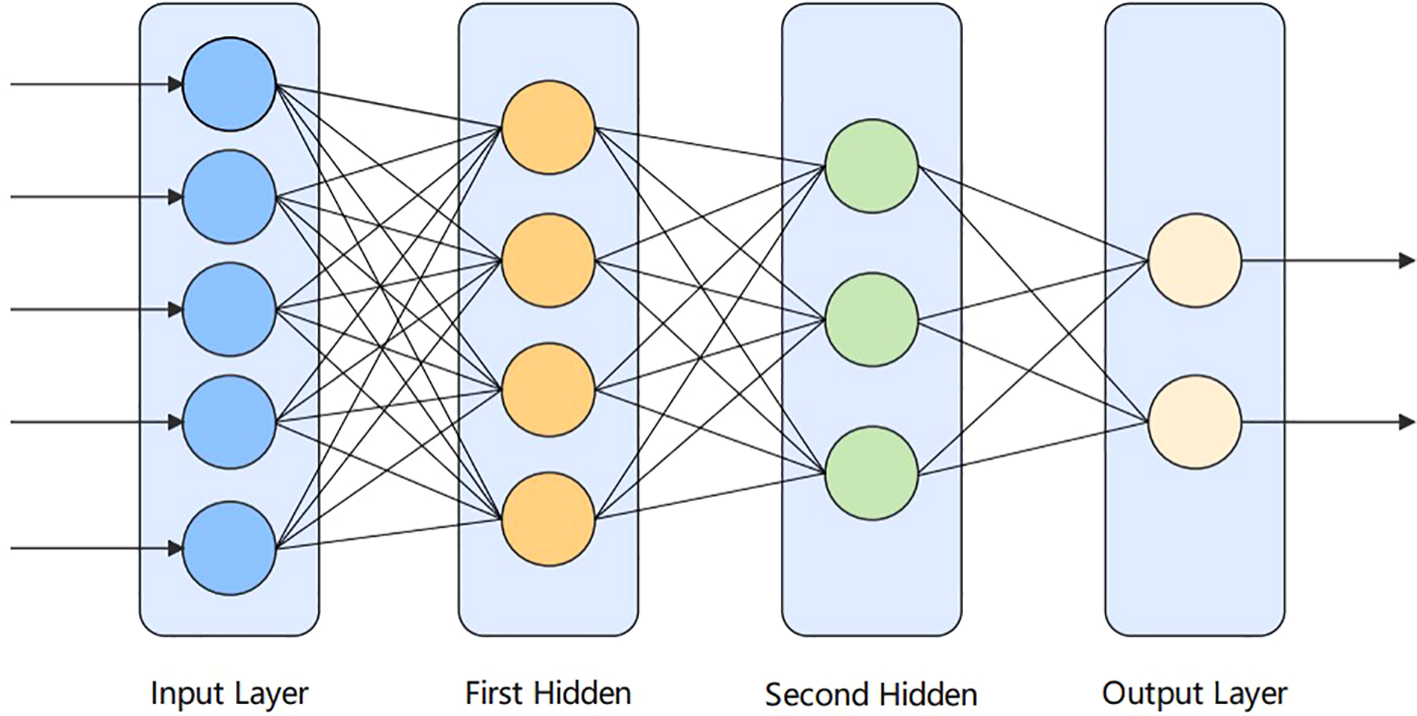
Artificial neural network diagram.

ANN, or networks of artificial neurons, refer to biologically inspired models that mimic the brain. ANN are widely used in cancer diagnosis. In the human brain, neurons are interconnected and transmit data to each other. It is similar to the interconnection of neurons in the human brain. Neural networks consist of a large number of artificial neurons, called units arranged in layer order. Having neurons in each layer and forming a complete network, these neurons are called nodes. It consists of three layers, which are: the input, hidden layer, and output layer. In this paper, the ANN model is applied containing two hidden layers in addition to the input and output layers. The structure diagram is shown in **Figure 3** below.

**Figure 4:**
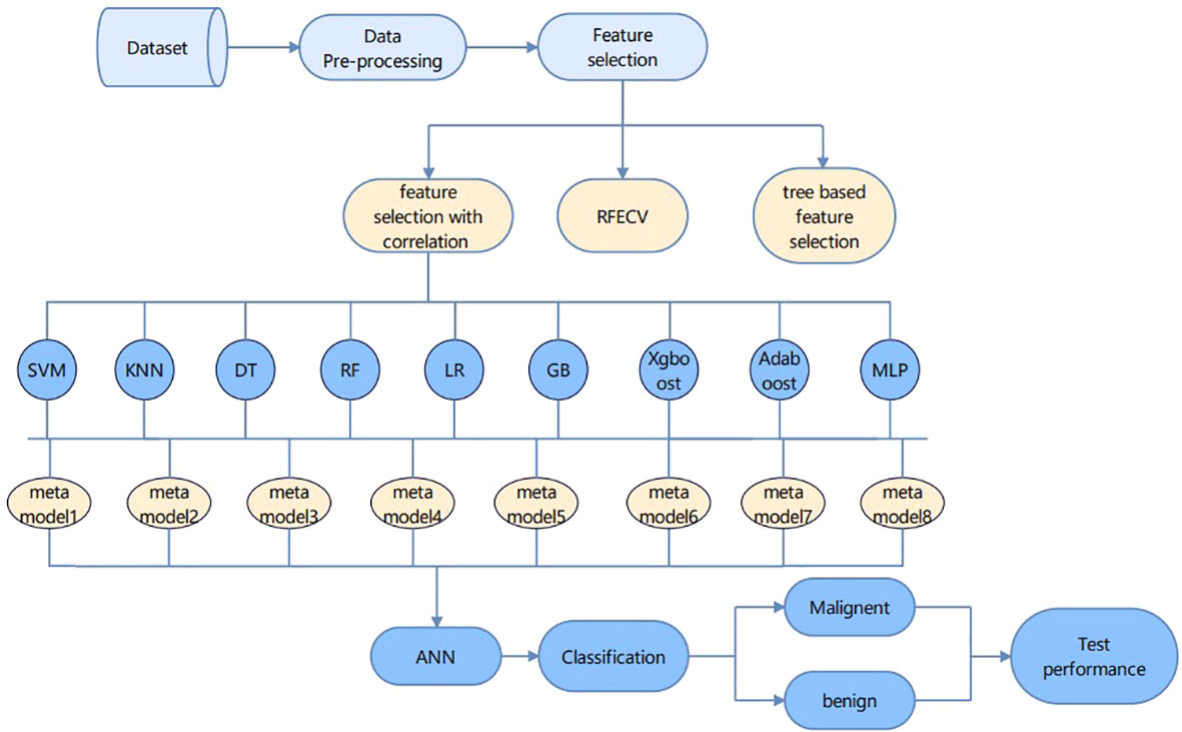
The overall research framework of the paper.

In the **Figure 4** framework diagram proposed in this paper, the relevance-based feature selection method is able to filter out the features that best represent all the information. Feature selection ends and enters the cancer classification and prediction part. Each learning model has its own advantages and disadvantages, and instead of choosing any one learning model, we try to build a meta-learning model to learn from learning, which will achieve better prediction by training a model on top of the previously trained model. The integration approach used in this paper is to first build several different types of base learners, such as Random Forest, KNN, SVM, etc., and use them to get the first level prediction results, and then build a meta-learner based on these first level prediction results to get the final prediction results. Eight meta models with an accuracy of more than 92% were selected, and the final result output is used as the input features of the ANN model to achieve high accuracy prediction of breast cancer.

### Performance evaluation metrics

In this paper, the classifier’s performance is measured using the evaluation metrics of the confusion matrix (13), in which four elements are included.

1. TM (True Malignant): The result shows that it is Malignant and the person does have Cancer.

2. TB (True Benign): The result shows Benign, the person is actually normal.

3. FM (False Malignant): The result shows Malignant, the person is actually normal.

4. FB (False Benign): The result appears in Benign and the person actually has cancer.

The confusion matrix is shown as an example in **Table 2**.

**Table 2:** Confusion matrix.

		Predicted	
		Malignant	Benign
Real	Malignant	True Malignant (TM)	False Benign (FB)
	Benign	False Malignant (FM)	True Benign (TB)


$$\begin{array}{c} Accuracy=\frac{TM+TB}{TM+FM+TB+FB}\left(3\right) \end{array}$$

$$\begin{array}{c} \mathrm{Pr}ecision=\frac{TM}{TM+FM}\left(4\right) \end{array}$$

Recall=TMTM+FB(5)

F1−score=2Precision×RecallPrecision+Recall(6)

## Results

In this paper, three feature selection methods are used, the feature selection method based on correlation has the highest accuracy of 96.49%, and the RFECV method selects too few feature values, which cannot fully contain the data information, as shown in the table. The feature selection method based on relevance takes into account the correlation between features and features, and finally 16 features are selected. As shown in **Table 3**.

**Table 3:** Evaluation metrics and results of feature selection algorithms.

Feature selection algorithm	Accuracy	Number of feature selection
**Correlation**	**0.9649**	**16**
RFECV	0.9649	12
Tree-based	0.9590	16

Bold represents the highest accuracy of the selected feature selection method.

In this paper, nine machine learning algorithms were used for breast cancer prediction accuracy and the models with accuracy greater than 92% were selected to create meta-learning models. The performance is shown below. As can be seen from **Table 4**, the meta-learning model results are input to the ANN model to get improved accuracy.

**Table 4:** Metrics and results of meta model evaluation of test data.

	Model	Accuracy	Precision	Recall	F1-score
1	Meta Model 1	0.9590	0.9117	0.9841	0.9465
2	Meta Model 2	0.9766	0.9538	0.9841	0.9687
3	Meta Model 3	0.9824	0.9687	0.9841	0.9763
4	Meta Model 4	0.9766	0.9538	0.9841	0.9687
5	Meta Model 5	0.9707	0.9393	0.9841	0.9612
6	Meta Model 6	0.9590	0.9117	0.9841	0.9465
7	Meta Model 7	0.9766	0.9538	0.9841	0.9687
8	Meta Model 8	0.9766	0.9538	0.9841	0.9687
**9**	**ANN**	**0.9874**	0.9538	0.9841	**0.9802**

Bold represents the highest accuracy and F1-score of the model, and the model has a good effect.

In **Table 5**, we compared the use of single classifier or hybrid model in other studies and found that the hybrid model of meta-learning and ANN proposed in this paper was able to achieve a good accuracy of 98.74%. Thus, it is also demonstrated that the hybrid model can achieve better accuracy results than the single model.

**Table 5:** Comparison with other classifiers.

Parameters	(29)	(30)	(17)	(22)	Proposed System
Models Used	Extreme Leaning+ Genetic Algorithm	DNN	LR, SVM, KNN hybrid XGBoost model	LR, ANN	Meta-Learning and ANN
Accuracy	97.28%	92.%	98.25%	98.5%	**98.74%**

Bold value represents the accuracy of the model used in this study in comparison with other models.

## Discussion

The framework of this paper contains two main parts: feature engineering, and the classification model. The relevance-based feature selection method with the highest accuracy is compared and the meta-learning models are created based on various supervised and unsupervised machine learning algorithms to obtain eight meta models with the best performance, and the performance metrics are mainly focused on accuracy. The stacking integration method is used for the eight meta-models, and the integration results are input to the ANN model to obtain. It can be seen that the performance index of the hybrid algorithm is greater than that of the individual algorithm, and the accuracy and F1-score are important indicators for evaluating the model prediction results. A hybrid meta-learning and ANN breast cancer prediction framework can improve prediction performance, obtaining 98.74% accuracy and 98.02% F1-score.

In the framework used in this paper, the feature engineering part can be optimized. Feature selection adopts the ensemble method to obtain better accuracy, by selecting the best feature to provide better input for the subsequent classification algorithm, to improve the accuracy of breast cancer classification. In the future, the framework of this paper can be applied to other cancer datasets with the expectation of achieving better performance in cancer diagnosis.

## Data availability statement

Publicly available datasets were analyzed in this study. This data can be found here: https://archive.ics.uci.edu/ml/datasets/Breast+Cancer+Wisconsin+%28Diagnostic%29.

## Author contributions

Conceptualization, LH and ZY. Writing, LH. Rresources, ZY. Project administration, ZY. Funding acquisition, ZY. All authors contributed to the article and approved the submitted version.

## Funding

This work was supported by National Natural Science Foundation of China (No:62072296)

## Acknowledgments

We would like to thank Chin-Shiuh Shieh and Yafei Dong for their constructive feedback on this manuscript.

## Conflict of interest

The authors declare that the research was conducted in the absence of any commercial or financial relationships that could be construed as a potential conflict of interest.

## Publisher’s note

All claims expressed in this article are solely those of the authors and do not necessarily represent those of their affiliated organizations, or those of the publisher, the editors and the reviewers. Any product that may be evaluated in this article, or claim that may be made by its manufacturer, is not guaranteed or endorsed by the publisher.
